# Use of the BRANT-MERQS scoring table for the quality assessment of type 3 medication review in patients with rheumatoid arthritis and those with type 2 diabetes mellitus

**DOI:** 10.3389/fphar.2024.1359568

**Published:** 2024-08-16

**Authors:** Anneleen Robberechts, Kaat Stas, Margot Puttemans, Laura Poppe, Stephane Steurbaut, Guido R. Y. De Meyer, Hans De Loof

**Affiliations:** ^1^ Meduplace, Royal Pharmacists Association of Antwerp (KAVA), Antwerp, Belgium; ^2^ Laboratory of Physiopharmacology, University of Antwerp, Antwerp, Belgium; ^3^ Centre for Pharmaceutical Research, Vrije Universiteit Brussel, Brussels, Belgium; ^4^ Department of Hospital Pharmacy, UZ Brussel, Jette, Belgium; ^5^ Infla-Med Research Center of Excellence, University of Antwerp, Antwerp, Belgium

**Keywords:** medication review, quality assessment, community pharmacy services, pharmaceutical care, community pharmacist, rheumatoid arthritis, type 2 diabetes mellitus, medication review tools

## Abstract

**Background:**

A type 3 medication review (MR3) is a patient-centred medication service primarily provided by pharmacists and is presently employed routinely in several countries. In this process, pharmacists interview patients and collaborate with the treating physician to optimize the patient’s pharmacotherapy, taking into account the patient’s medication history and other medical data including laboratory values. The need to maintain the quality of such interventions during and after their initial implementation cannot be overstated.

**Aim:**

The objective of this study was to refine and assess a scoring table to evaluate the quality of MR3 conducted in Belgian community pharmacies.

**Methods:**

The comprehensive quality of MR3s was assessed by scoring its various components using a previously developed scoring table, called BRANT-MERQS, Brussels Antwerp Medication Review Quality Score. MR3s were analysed from an implementation study with patients suffering from rheumatoid arthritis (RA, subproject 1) and type 2 diabetes mellitus (T2DM, subproject 2). Additional information was obtained during a telephone call with a subset of participating pharmacists of subproject 1 who finalized their first MR3.

**Results:**

In subproject 1, a total of 21 MR3s of patients with RA were examined. The assessment showed favourable scores for elements such as a well-organized medication schedule, treatment adherence, and the elaboration of specific interventions. However, certain other quality criteria posed challenges in the evaluation, for example, the use of simple and understandable language. Pharmacists faced time constraints, and elderly general practitioners (GPs) displayed limited enthusiasm, which were notable barriers observed for this subproject. In the context of subproject 2 that investigated 41 MR3s in patients with T2DM, the quality criteria of interaction between pharmacist and GP, and used sources and tools received high scores. However, there was still room for improvement, especially in areas such as accurate dosing, handling kidney function, QT prolongation, correctly associating laboratory values with relevant drugs and medical conditions, and optimisation of medication schedules for patients.

**Conclusion:**

This study demonstrated the feasibility of MR3 quality assessment through a scoring system. However, it also unveiled the tool’s current imperfections and highlighted the ongoing need for refinement, something expected of a new service in an implementation phase.

## 1 Introduction

Quality assurance plays a crucial role in ensuring the long-term success and effectiveness of a newly implemented service. By implementing robust quality assurance measures, organizations can proactively identify and address many potential issues or shortcomings, thus enhancing the overall quality and reliability of the service. This not only instils confidence in both service providers and health insurance companies but also helps to establish a solid foundation for continuous improvement and innovation, while improving patient safety. By upholding high standards through quality assurance, organizations can strive for excellence and deliver a service that meets or exceeds expectations, fostering positive patient experience and satisfaction, and the long-term sustainability of the service (World Health Organisation, 2018). Already in 1969, a guideline from the Committee for National Health Insurance of the United States of America, stated that the national health insurance program should encompass provisions aimed at ensuring the quantity, quality, effectiveness, continuity, and cost-efficiency of the family healthcare services it supported (Weinerman, 1971). More recently, the Interactive Systems Framework for Dissemination and Implementation (ISF) acknowledges the critical role of quality assessment in widespread innovation success (Wandersman et al., 2008).

Since 2017, the Royal Pharmacists Association of Antwerp (KAVA) has overseen initiatives related to type 3 medication review (MR3) in Belgium. Type 3 medication review, as classified by The Pharmaceutical Care Network Europe (PCNE), involves a thorough assessment of a patient’s medication, starting with a comprehensive medication history, incorporating medical data, and involving an extensive patient interview along with feedback from the general practitioner (Griese-Mammen et al., 2018; Robberechts et al., 2021; Robberechts et al., 2024). The objective is to optimize the patient’s pharmacotherapy while minimizing potential medication errors, reduce waste, and enhance medication adherence (Griese-Mammen et al., 2018). KAVA’s initiatives involved training pharmacists for MR3 and guiding them to implement it effectively in their community pharmacies (Robberechts et al., 2021). Responding to requests from participating pharmacists in the pilot initiative, additional courses were introduced in 2021 and 2022. These courses, tailored to pharmacists with prior MR3 training, focused on specific patient populations, with the 2021 course addressing rheumatoid arthritis (RA, subproject 1) and the 2022 course centring on patients with type 2 diabetes mellitus (T2DM, subproject 2).

Rheumatoid arthritis is a chronic, systemic, inflammatory autoimmune disorder. The condition is linked to substantial morbidity and mortality risks (Akil and Amos, 1995). While scientific research on medication review (MR) in patients with RA is limited, some existing studies centre around medication adherence, drug-related problems (DRPs) and pharmaceutical interventions within this patient group (Neame and Hammond, 2005; Joplin et al., 2015; Yailian et al., 2019; Chang et al., 2021). The clinical significance of pharmaceutical interventions during MRs was examined in 2018 in the rheumatology department of a French hospital (Yailian et al., 2019). Using MR type 2b, i.e. starting from clinical data but without active patient input (Griese-Mammen et al., 2018), the study uncovered a considerable number of DRPs of substantial clinical relevance. Effective collaboration between pharmacists and physicians led to the necessary pharmaceutical interventions (Yailian et al., 2019).

Another major chronic condition is T2DM. Despite its potential for various complications, adherence to pharmacological treatment for T2DM is often suboptimal (Donnan et al., 2002). Moreover, effective lifestyle modifications are frequently lacking. Despite the crucial role of pharmacists in primary care, they are not consistently involved in the follow-up care of patients with T2DM (Blenkinsopp and Hassey, 2010). Nonetheless, research has demonstrated the significant impact of pharmacists in providing advanced care for patients with multiple chronic diseases (Latif, 2017; Rosli et al., 2021).

Various studies have demonstrated that MR positively impacts glycaemic control, quality of life, medication adherence, lifestyle adjustments, disease understanding, and the rate of DRPs (Lindenmeyer et al., 2006; Blenkinsopp and Hassey, 2010; Ko et al., 2016; Schindler et al., 2020; Rosli et al., 2021). Common DRPs include poor adherence, inappropriate drug selection, contraindications and side effects. Additionally, enhanced understanding of conditions and improved adherence have been linked to better glycaemic control (Lindenmeyer et al., 2006; Blenkinsopp and Hassey, 2010; Rosli et al., 2021).

In recent years, a limited but growing emphasis on the quality dimension of MRs can be recognized (Harding and Wilcock, 2010; Krska et al., 2010; Mestres Gonzalvo et al., 2014; Castora-Binkley et al., 2023). Standards and evidence-based guidelines must guide MRs. This is crucial for assisting pharmacists and the broader clinical pharmacy team in achieving optimal results for both patients and the healthcare system (Paudyal et al., 2023). In Canada, a study outlined that community pharmacists, when implementing a reimbursed MR program, prioritized strategies emphasizing service efficiency and quantity rather than quality (MacKeigan et al., 2017). With regard to prioritizing quality, our previous research identified eight key elements in assessing MR3 quality (Robberechts et al., 2023a). These components involve using clear language, explaining the review’s purpose, addressing medication adherence, discussing specific drug use, creating and reviewing a comprehensible medication schedules with the patient, assessing ongoing relevance of all drug indications, providing an opportunity for patients to discuss their symptoms with the pharmacist, and considering patient expectations and concerns in treatment planning (Robberechts et al., 2023a). The current study has incorporated these findings, yet the primary objective is not to replace future Patient-Reported Outcome Measures (PROMs) and Patient-Reported Experience Measures (PREMs) but rather to conduct a quality assessment of the content within MR3 reports.

The objective was to further develop and evaluate a scoring table to assess the quality of MR3, focusing on its application in patients with RA and T2DM (Robberechts et al., 2023a).

Ethical approval was granted by the UZA/University of Antwerp medical ethics committee in September 2020 with authorization number 20470 20200921 DGA UZA.

## 2 Materials and methods

### 2.1 Data collection

This study consisted of two subprojects: one focused on patients with RA (subproject 1) and the other on patients with T2DM (subproject 2). Pharmacists who participated in prior MR3 training sessions were invited by email. The outcomes were processed on a per-pharmacy basis, considering that pharmacists were sometimes collaboratively conducting MR3s within the same pharmacy. Subproject 1 was conducted between September 2020 and June 2021, while subproject 2 took place from March 2022 to April 2023. At the beginning of each subproject, pharmacists were provided with customized Supplementary Material, including a locally adapted step-by-step medication assessment guideline based on the one developed by the Royal Dutch Society for the Advancement of Pharmacy (KNMP), instructions for calculating medication adherence, model letters for general practitioners (GPs) and patients, a prototype for obtaining informed consent from patients, and a review report template (Koninklijke Nederlandse Maatschappij ter bevordering der Pharmacie, 2013). Furthermore, participating pharmacists underwent specific training and received ongoing support and guidance from the research team (A.R. and H.D.L.) throughout the project’s duration (Robberechts, 2019).

Pharmacists who provided anonymised MR reports to the research team were given a minor financial reward, as MR3s were not yet reimbursed in Belgium during that period. These reports were used to assess the MR3 quality. Participants in subproject 1 could employ either a Word template or a Google Forms to generate their reports. Subproject 2 adopted a consistent reporting method and used only the Google Forms, reflecting the preference of 71% of those involved in subproject 1. The Google Forms utilized for both projects was identical, except for the questions about the particular project (RA or T2DM). All templates can be found in the appendix.

### 2.2 Design of the study

The evaluation of MR3 quality assessment was conducted and documented using a scoring table, called BRANT-MERQS, Brussels Antwerp Medication Review Quality Score, as showed in Table 1. To reduce bias, researchers independently evaluated predetermined quality criteria without disclosing the scoring table’s content to the pharmacists. The table was created using quality criteria derived from prior research (Robberechts et al., 2023a), which identified broad consensus-based key elements for assessing MR3 quality. In addition, the hierarchy of quality assessment criteria for MR3s was also widely agreed upon (Robberechts et al., 2023a). The scoring table was therefore structured into six themes: 1) general aspects, 2) drug assessment, 3) treatment evaluation, 4) consultation between pharmacist and GP, 5) sources and tools, and 6) in-depth analysis of RA or T2DM. In the context of subproject 1, a comprehensive assessment of 46 quality criteria was conducted. For the second subproject, limited modifications were made to the quality criteria used in the first subproject by incorporating an additional three quality criteria specific to T2DM. This resulted in a cumulative total of 48 quality criteria being evaluated in the second subproject.

**TABLE 1:** The BRANT-MERQS scoring table used for the two subprojects.

BRANT-MERQS scoring table		Included in total score
General quality criteria	Explanation benefit and purpose of MR	No
	General characteristics (birth date, gender)	Yes
	Allergies and intolerances	Yes
	Lab values	Yes
	Current conditions	Yes
	Simple and comprehensible language	No
	Familiar and quiet environment	No
Quality criteria of the drug treatment	Overview chronic medication	Yes
	Overview self-care medication	Yes
	Indications known by patient	No
	Medication schedule recent	Yes
	Indications still topical	No
	Effectiveness	Yes
	Side effects	Yes
	Drugs treating side effects	No
	Interactions with other drugs/food	Yes
	Relevance of interactions	Yes
	Correct dose	Yes
	Vaccination status	Yes
	User-friendly administration form	Yes
	Control of storage of medication	No
Quality criteria of the current overall treatment	Treatment construction	No
	Treatment choice as a function of comorbidities	No
	Drug changes + motivation	No
	Nonmedical measures	No
	Undertreatment	Yes
	Overtreatment	Yes
	Tapering of medication	No
	Addiction risk	No
	Adherence	Yes
	Motivation control by healthcare providers (HCP)	Yes
	Interpretation lab values	Yes
	Lab values linked to conditions and drugs	Yes
	Tools: medication schedule, medication box	Yes
Quality criteria of the interaction between pharmacist and GP	Report to GP	Yes
	Elaboration of specific interventions	Yes
	Interventions sufficiently reasoned	Yes
	Intervention plan discussed with patient	No
	Actions without consulting GP (non-medicinal)	No
	Actions after consultation of GP	No
	Follow-up interview with the patient	No
Quality criteria of the used sources and tools	Availability of lower cost alternatives	No
	Use of reliable tools	Yes
	Bibliography	Yes
	Reliable literature	Yes
Project specific quality criteria	Accurately estimate osteoporosis risk using the fracture risk assessment (FRAX) tool and interpret the results correctly	Subproject 1
	HbA1c	Subproject 2
	HbA1c target known?	Subproject 2
	Is the patient part of a care program?	Subproject 2

During subproject 1, pharmacists who finalised their first MR3 were contacted by phone offering support during this early implementation phase between March and April 2021. Furthermore, they were reminded about any incomplete data and were asked some general questions to inform the overall subproject implementation. This was not the case for subproject 2.

### 2.3 Data analysis

The same scoring table was employed to assess each aspect of the MR3 report for patients with RA or T2DM and consisted of the following ratings: very good (3), good (2), insufficient (1), and not present in the report (0). As an illustration, a score of 0 was assigned when no laboratory values were included in the report. In instances with incomplete values, such as a missing important parameter, a score of 1 was given. A score of 2 was attributed to a rather comprehensive list of laboratory values with room for improvement for example, the missing of a less important laboratory value, while a score of 3 was assigned when no further enhancements were deemed necessary. The total score was obtained by adding up the individual scores from all assessable statements. The total score comprised only the quality criteria that were quantifiable. The highest possible total score for subproject 1 was 84, and for subproject 2, it was 90. To enhance interpretability, the results were transformed into percentages. Other quality criteria were not measurable as they could not be evaluated solely through a written report, like, for instance, elucidating the benefits and purpose of MR.

In subproject 1, K.S. conducted the data analysis, and in situations where uncertainties arose, she reached out to A.R. and H.D.L. For subproject 2, L.P. and M.P. were responsible for the data analysis. Similarly, in case of any uncertainties, they consulted A.R. and H.D.L. for assistance.

## 3 Results

Using the scoring table, an aggregate score was determined by summing up individual scores for each measurable quality criterion. General criteria with insufficient reporting or non-measurable aspects were excluded from the total score calculation, accounting for 18 criteria in both subprojects. The distribution of the scores can be found in the attachment.

### 3.1 General results subproject 1: patients with RA

**FIGURE 1:**
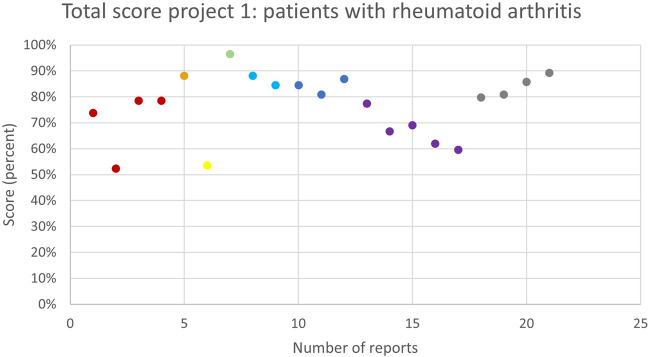
The overall quality assessment scores (in %) for each MR3 report of subproject 1 carried out by the same pharmacy team are shown in the same colour. Different colours represent different pharmacy teams.

52 pharmacists who previously participated in trainings around MR3, were invited to participate in the first subproject and garnered participation from fifteen pharmacies. Out of these, eight (53%) individual pharmacies ultimately conducted at least one MR3, as detailed in Table 2. All pharmacies were located in the province of Antwerp and a total of 21 MR3s were collected. The quality assessment of these MR3s was analysed and described using the score table provided in the appendix. The average total score was 65 (77%). The highest score of the 21 MR3 in this subproject was 81 (96%) and the lowest score was 44 (52%), as illustrated in Figure 1.

**TABLE 2:** General characteristics of the two subprojects.

	Subproject 1: RA	Subproject 2: T2DM
Timing	September 2020 – June 2021	March 2022 – April 2023
Participating pharmacies who submitted at least one MR3	8	7
Total MR3 reports	21	41
Patients’ characteristics	29% male patients, age between 69 and 85; median = 76 71% female patients, age between 32 and 90; median = 64	54% male patients, age between 56 and 91; median = 69.5 46% female patients, age between 49 and 89; median = 70
Average score of the MR3 reports (%)	77%	67%
Highest score of the MR3 reports (%)	96%	86%
Lowest score of the MR3 reports (%)	52%	40%

### 3.2 General results subproject 2: patients with T2DM

Subproject 2 extended invitations to 114 pharmacists who had undergone prior training on MR3. Eighteen pharmacies participated in the T2DM subproject. Eventually, seven pharmacies (38%) performed at least one MR3. Compared to subproject 1, these pharmacies were distributed across a broader geographic area in Flanders, with four in the province of Antwerp, one in Flemish Brabant, one in Limburg, and one in East Flanders. Together, 41 MR3 reports were completed and could be analysed.

The quality assessment of these reports was evaluated using the scoring table that was slightly adapted to the context of T2DM (see Table 1). According to the medical history obtained from the GP, the patients suffered from three chronic conditions on average, including T2DM.

**FIGURE 2:**
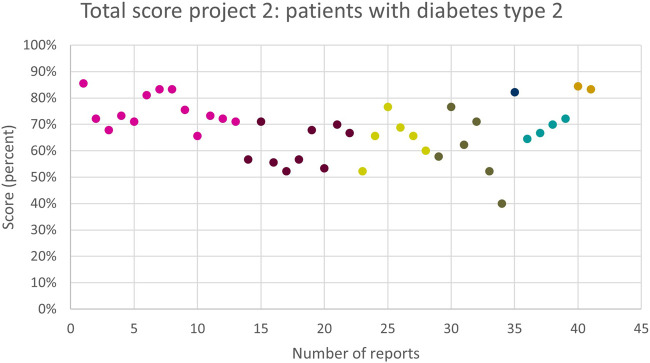
The overall quality assessment scores (in %) for each MR3 report of subproject 2 carried out by the same pharmacy team are shown in the same colour. Different colours represent different pharmacy teams.

The total score per MR3 is shown in Figure 2. The average total score of the 41 reports was 61 (67%). The highest score given to a MR3 in this subproject was 77 (86%), as shown in Figure 2. The lowest score was 36 (40%). The total score of some, but not all, pharmacies improved as they performed more MR3s. Several pharmacies showed strong variability and no obvious trend. Two pharmacies, displayed in dark blue and orange, performed two and one MR3(s), respectively, but scored remarkably high.

### 3.3 Scoring table BRANT-MERQS

Table 3 presents the quality criteria of BRANT-MERQS that consistently achieved the highest scores in both subprojects.

**TABLE 3:** Quality criteria for subproject 1 and 2 with the highest scores.

Quality criteria	Quality criteria in tool
Patient’s general characteristics were carefully documented	General characteristics (birth date, gender)
The pharmacist conducted a comprehensive review of the patient’s chronic medication	Overview chronic medication
A recent and well-structured medication schedule was included	Medication schedule recent
Side effects were taken into consideration	Side effects
Overtreatment was thoroughly examined	Overtreatment
Patient medication adherence was diligently assessed by the pharmacist, using a table provided for calculations	Adherence
The use of tools or devices such as medication schedules, medication boxes, and pill cutters was inquired about	Tools: medication schedule, medication box
Use of reliable tools such as START – STOPP criteria and GheOP³s tool O’Mahony et al. (2015); Foubert et al. (2021)	Use of reliable tools
Use of reliable literature such as local and international guidelines	Reliable literature

#### 3.3.1 General quality criteria

Out of the seven quality criteria that constituted the first theme, four could be determined directly from the report: the patient’s general characteristics (date of birth, gender), allergies or intolerances, presence of laboratory values, and current conditions, as presented in Table 1. The remaining three quality criteria include the explanation of MR3s purpose and usefulness to the patient, the use of simple and understandable language with the patient and ensuring a familiar and calm environment during the consultation.

**FIGURE 3:**
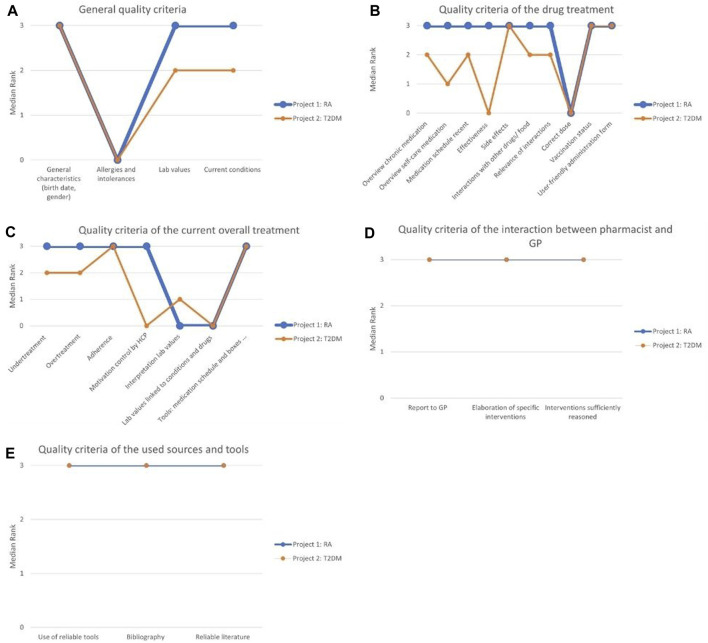
**(A)** The distinctions and similarities between the two projects for the general quality criteria; **(B)** The distinctions and similarities between the two projects for the quality criteria of the drug treatment; **(C)** The distinctions and similarities between the two projects for the quality criteria of the current overall treatment; **(D)** The distinctions and similarities between the two projects for the quality criteria of the interaction between pharmacist and GP; **(E)** The distinctions and similarities between the quality criteria of the used sources and tools. RA, rheumatoid arthritis; T2DM, type 2 diabetes mellitus.

The patient’s general characteristics was consistently reported by all pharmacists and scored “very good” across all reports, as shown in Figure 3A. Similarly, the collection of data such as laboratory values and current conditions scored well, with most reports describing this aspect as “good” or “very good”. In subproject 1, 91% received a score of “good” or “very good” for this aspect, while in subproject 2, the corresponding percentage was 73%. However, in a few MR3s, these elements were either overlooked or lacked sufficient data. For example, in subproject 2, twelve MR3 reports (29%) received an “insufficient” score regarding current conditions as more than half of the drugs in the medication schedule were not linked to a current condition provided by the GP. This occurred because the pharmacist initially prepared the medication schedules without knowledge of the actual conditions and no optimisation of the regimens took place after having gained insights in the patient’s conditions following the MR3s. Notably, the inquiry about allergies or intolerances was mostly missing from the reports (52% in subproject 1, 85% in subproject 2), although eight MR3s of subproject 1 (38%) addressed it appropriately and received the highest score.

#### 3.3.2 Quality criteria of the drug treatment

The second theme included all quality criteria related to a patient’s medications, with various aspects such as an overview of chronic medication, effectiveness, side effects, ease of use and vaccination status. Table 1 presents the corresponding fourteen quality criteria. Out of these, ten quality criteria were considered quantifiable from the reports and were integrated into the assessment of the MR3 reports, as illustrated in Figure 3B.

For the quality criteria concerning side effects, vaccination status and user-friendly medication administration, both subprojects scored “very good”. Two quality criteria, the overview of chronic medication and its inclusion in a medication schedule, were present in almost all MR3 reports. In subproject 1, every report (100%) received a “good” or “very good” score for the overview of chronic medication and its inclusion in the medication schedule. However, in subproject 2, the corresponding percentages were 76% for the overview of chronic medication and 81% for inclusion in the medication schedule. The lower score in subproject 2 was attributed to incomplete information.

Concerning drug interactions and their importance to the patient, the majority of MR3 reports garnered favourable ratings of “good” or “very good”. In subproject 1, 76% received a score of “good” or “very good” for interactions with other drugs or food, and 81% of the reports were rated “good” or “very good” regarding the relevance of the interactions. In subproject 2, these percentages were 68% and 61%, respectively.

The evaluation of self-care medications, dietary supplements and homeopathy received mostly positive scores in subproject 1 but scored less well in subproject 2. In subproject 1, 86% of the reports were rated “good” or “very good”, whereas in subproject 2, this was only 46%.

Pharmacotherapeutic effectiveness reporting was not included in six reports (29%) from subproject 1 and in 31 reports (76%) from subproject 2. However, in subproject 1, the remaining 15 reports (71%) effectively addressed this aspect and received the highest score, while this was only the case for 5 reports (12%) in subproject 2. The verification of the patient’s drug dosage was infrequently reported in both subprojects.

Furthermore, specific quality criteria were excluded from the scoring table due to measurement complexity or because the reports did not explicitly address them, leading to insufficient data collection for these criteria. These included verifying whether the patient was informed about the indications for each medication, ascertaining the relevance of these indications, checking for medications used to address side effects, and ensuring the proper storage of medications.

#### 3.3.3 Quality criteria of the current overall therapy

The third theme covered thirteen quality criteria pertaining to the patient’s specific treatment that included aspects such as therapy appropriateness and choice, under- and over-treatment, therapy adherence, addiction risk, and motivation for regular physician visits. Seven of these criteria were deemed sufficiently measurable and were integrated into the assessment of the MR3 reports, as outlined in Table 1 and Figure 3C.

The quality criteria that scored best were related to the tools used for medication management, such as medication schedules, pill cutters and medication boxes, as well as therapy adherence, present in almost every report. For subproject 1, 95% of the reports scored “good” or “very good” for the quality criteria regarding tools, medication schedule and medication boxes, and for subproject 2 this was 100%. For adherence, this was 100% for the first subproject and 76% for the second subproject.

In terms of under-treatment and over-treatment, the majority of MR3 reports received favourable ratings of “good” or “very good”. For under-treatment, this constituted 81% in subproject 1% and 73% in subproject 2, while for over-treatment, the percentages were 95% in subproject 1% and 78% in subproject 2. However, in subproject 2, nine reports (22%) were assessed as “insufficient” for not adequately addressing under-treatment and over-treatment.

The quality criteria regarding patient check-ups with various healthcare providers (HCP) resulted in positive scores in subproject 1, with 86% scoring “very good”, but was mentioned less frequently in subproject 2, where only 32% of MR3 reports scored “very good”.

Conversely, there was greater variability and a frequent absence of two other quality criteria in the reports. Specifically, the accurate interpretation of laboratory values was absent in 57% of reports in subproject 1 and 49% in subproject 2, and their correlation with drugs or medical conditions was absent in 67% of reports in subproject 1 and 56% in subproject 2.

The quality criteria relating to appropriate therapy choices based on comorbidities, non-drug measures, and addiction risk could not be adequately assessed based on the data provided by the pharmacists. Additionally, several quality criteria within this theme were only found in a limited number of MR3 reports.

#### 3.3.4 Quality criteria of the interaction between pharmacist and GP

The fourth theme explored seven components of the pharmacist’s interaction with the GP, encompassing the communication with the GP and the validation of interventions. Three quality criteria were considered quantifiable to a satisfactory degree and were incorporated into the analysis of the MR3 reports, as delineated in Table 1 and Figure 3D.

All evaluable quality criteria within this theme scored quite high in both subprojects. These criteria encompassed the GP report, the detailed explanation of specific interventions, and the appropriateness of the reasoning for the interventions. In subproject 1, 72% of the reports received a rating of “good” or “very good” for the report to the GP, whereas in subproject 2, this percentage was 86%. Regarding the initiation of specific interventions, 95% received a score of “good” or “very good” in subproject 1, and 85% in subproject 2. For quality criteria regarding the appropriateness of the reasoning for the interventions, 91% received a score of “good” or “very good” in subproject 1, while the corresponding percentage for subproject 2 was 68%.

Unfortunately, the quality criteria concerning the intervention plan developed in collaboration with the patient, the actions taken post-consultation with the GP and the follow-up interviews with the patient could not be evaluated using the scoring table due to an insufficient amount of data from the pharmacists’ reports.

#### 3.3.5 Quality criteria of the used sources and tools

The fifth theme centred on four quality standards associated with the resources, guidelines, and tools employed by pharmacists during the MR3 process. These standards encompassed the use of reliable tools, the incorporation of a bibliography, the trustworthiness of the cited literature and the identification of cost-effective medication alternatives for patients.

In the overall assessment, the first three criteria factored into the total score, and each of them demonstrated high performance in both subprojects, as presented in Figure 3E. In the first subproject, 19%, 14%, and 19% of the reports respectively did not include these three quality criteria. In contrast, in the second subproject, all quality criteria related to sources and tools were always present. The second subproject displayed minimal variation in results, with scores predominantly falling within the range of “good” to “very good”.

However, the criterion related to the identification of cost-effective alternatives could not be evaluated in both subprojects since it was mentioned in only one report, rendering it impossible to assign a score.

#### 3.3.6 In-depth analysis of RA and T2DM

The sixth and final theme centred on the specific conditions of both subprojects. For the RA subproject, the sole component in this theme was to accurately estimate osteoporosis risk using the Fracture Risk Assessment (FRAX) tool. The tool is a valuable resource for estimating an individual’s fracture risk (Vandenput et al., 2022) and was used in 17 (81%) reports resulting in a “good” or “very good” score.

Regarding the T2DM subproject, only two (5%) MR3 reports did not receive the maximum score of “very good” on the item related to the patient’s HbA1c. One report did not mention it at all, while the other MR3 report provided an interpretation of the HbA1c value as “to high”, leading to a score of “good” since it was based on an interpretation rather than the presence of the precise value. Most MR3 reports scored “very good” on reporting the item “HbA1c target known”, except for two reviews (5%) that failed to mention it. All MR3 reports received a score of “very good” on the item “patient in a care program”. Among the 21 patients (51%) enrolled in a care program, eight patients were aware of their target HbA1c value. In contrast, only 2 out of the 20 patients who were not in a care program knew the appropriate target value for their HbA1c.

### 3.4 Results of the phone conversation regarding subproject 1

The phone conversations with the seven pharmacists who had submitted at least one MR3 between March and April 2021 yielded positive and enthusiastic feedback.

#### 3.4.1 Overall appreciation of subproject 1

Feedback on this first subproject was generally positive. The COVID-19 crisis was frequently cited as the primary cause of delays in performing medication reviews. Some pharmacists mentioned challenges in collecting data from the GP and contacting patients. However, they found the model letter for physicians to be a helpful tool. Utilizing a single theme, RA, proved to be an efficient approach for conducting multiple MR3s. However, recurring concerns emerged about the intensive preparation and time-consuming nature of the MR3s. Despite the acknowledged challenges, pharmacists recognized the potential of MR3s to provide significant benefits to patients.

#### 3.4.2 Materials used for the reports

Pharmacists were asked about their preferred method of reporting MR3s and a majority opted for the Google Forms. They valued its convenience for updating the report at any time. However, some pharmacists found it more challenging to input complex information, such as medication schedules, laboratory values and adherence tables in the Google Forms.

#### 3.4.3 Questions or support needed

When pharmacists were asked about their need for additional support, three of them asked for clarification on the interpretation of laboratory values. They expressed uncertainty about the appropriate course of action based on the results. One pharmacist requested more specific content for the model letters addressed to both the physician and patient, while another proposed providing a concise report for the patient after the consultation. Additionally, several pharmacists repeatedly asked for an extension of the subproject’s deadline.

#### 3.4.4 Feedback from GPs

Pharmacists mentioned that GPs expressed positive feedback regarding the subproject. Although one GP initially had difficulty grasping the concept, all eventually embraced it. In the majority of instances, discussions with GPs about possible actions resulting from the MR3s proceeded without difficulty. However, according to the pharmacists, a few GPs retained a degree of ambiguity or generality when deliberating about specific follow-up interventions.

#### 3.4.5 Time investment

There was a significant variation in the responses regarding the time needed for the preparation of MR3, conducting patient interviews, and processing data. The time spent on preparation ranged from 30 min to 15 h, while patient interviews usually lasted between 30 min and 1.5 h. Data processing and creating the report could vary from one to 8 h. Pharmacists expressed their anticipation that future reviews could be conducted somewhat more quickly.

## 4 Discussion

To guarantee effective quality assessment and support future implementation, it is imperative to conduct an analysis of the quality of individual MR3 reports. This study explored the feasibility of a specific framework for integrating quality assessment into the design of this complex intervention. Earlier research has underscored the importance of various key elements in ensuring MR3 quality (Robberechts et al., 2023a), and this study examined the applicability of these findings by testing them in two distinct practice-based projects that were part of an overall implementation initiative (Robberechts et al., 2021; Robberechts et al., 2023b).

The cumulative scores of individual reviews offer a comprehensive estimate of the MRs’ quality. The ratings generally ranged from “good” to “very good”, although it’s noteworthy that both positive and negative outliers existed. However, the decision on the benchmark score for an acceptable quality level of MR3, or the present need for establishing that value, remains to be addressed. Nevertheless, this study allowed to assess the feasibility of developing such a score and to accumulate the experience needed to inform future developments.

The findings of this study indicate that certain pharmacy teams progressed in the quality of their MR3s, likely by establishing a uniform implementation approach and the accumulation of expertise within a subproject. Conversely, some scores modestly declined, potentially linked to variations in pharmacists’ proficiency within the team or the intricacies associated with subsequent and potentially more complex cases (Robberechts et al., 2021).

The assessment of the reports also varied somewhat depending on the graders involved. In the second subproject, the evaluation process was more stringent compared to the first subproject, underscoring the necessity for a comprehensive scoring manual providing clear guidance. The first subproject’s higher scores may be attributed to participants receiving reminders about incomplete data during telephone conversations. It is also possible that a difference in multimorbidity between the patients in the two subprojects has an impact on the quality assessment scores, in addition to a difference in involvement of specialist care. Regardless, the objective remained analysing the utility of the scoring table for evaluating the quality of MR3 reports as part of assessing the quality of this pharmaceutical care service.

Within the MR3 reports, certain elements received suboptimal or no scores. Despite the acknowledged importance of these aspects, they were at times inadequately covered or not included in the examined reports (Robberechts et al., 2023a). The areas where scores were suboptimal or incomplete mainly involved accurate dosing, handling kidney function decline, QT prolongation, and correctly associating laboratory values with the relevant medications and medical conditions. While the participants received training on these topics, it may require more practice or additional training to become adequately experienced in it. These difficulties were not a surprise in the light of a recent study that documented the difficulties in handling QT interval pop-ups in a larger cohort of community pharmacists in Belgium (Bogaerts et al., 2023). Undoubtedly, these aspects will need to be integrated into future implementation and training programs.

The remaining criteria that lacked sufficient scores or had to be excluded typically pertained to elements that were either not assessed or found in only one of the two report templates. This absence of certain statements in the uploaded reports suggests that there is room for improvement in the design of the report format. Examples of such criteria include discussions about the usefulness and purpose of a MR with the patient, the use of simple and understandable language by the pharmacist during interaction with the patient, awareness of the reason for medication use by the patient, and verification of the necessity of all medications. Previous research has highlighted the significance of these criteria (Robberechts et al., 2023a). This limitation in the process can only be addressed if there is a willingness to allocate both time and resources for the random surveying of patients (Fraeyman et al., 2017). Additionally, follow-up is essential, but assessing the quality criteria for the intervention plan, post-consultation actions with the GP and patient follow-up interviews were hindered by insufficient data in the pharmacists’ reports.

The statement about allergies and intolerances was only present in the Word template contributing to the lower score for this criterion in most of the MR3s. Further optimalisation of the report form is therefore required. Striking a balance between the completeness of the report form and its practical feasibility is essential. Effective digital tools, such as intelligent decision support systems, have the potential to improve the efficiency and quality of MRs (Bindoff et al., 2007; Maierhöfer et al., 2022; Dabidian et al., 2023). Additional variation in the reports arises from the fact that variables such as addiction risk, abrupt medication adjustments, medication tapering, and non-drug interventions are not applicable to every patient.

MR3s were not yet a routine procedure and in subproject 1 the pharmacists valued the feedback obtained during the telephone conversations which aided them in enhancing their review quality and adjusting to this novel service. They also frequently cited the COVID-19 crisis causing delays in performing the reviews.

To the best of our knowledge, this study is a first attempt to assess the quality of MR3s using a scoring system offering a transparent view of pharmacists’ actions during MR3. However, there is room for further development. A limitation of the first subproject was the availability of two reporting options hampering exhaustive comparison of the scoring. In both subprojects, starting from the anonymized reports, it is not possible to gauge all aspects of the MR3s such as the assessment of plain and comprehensible language with the patient. This points to the need for the potential inclusion of PROMs/PREMs to comprehensively assess the quality of the MR3 process (Robberechts et al., 2023a). Earlier research shows that PREMs effectively assess older patients’ experiences with deprescribing in hospitals and can evaluate interventions to improve awareness, shared decision-making, and information provision (Ngui et al., 2023).

Another limitation was the possible inconsistency in the researchers’ evaluations because in subproject 1 a single researcher conducted the report analysis, consulting senior researchers when uncertainty arose, while in subproject 2, two researchers with the option to seek guidance from senior researchers were involved. A manual that guides a uniform scoring process would be advantageous for future uses.

We could only retrieve a limited number of studies about assessment of MR quality (Shrank et al., 2007; Harding and Wilcock, 2010; Krska et al., 2010; Mestres Gonzalvo et al., 2014; Beuscart et al., 2018; Livet et al., 2019; Rose et al., 2020; Clements et al., 2021; McCahon et al., 2021; Schröder et al., 2023). Nonetheless, there are indications of an uptake in the evaluation of pharmaceutical care quality, particularly in settings like nursing homes (Damiaens et al., 2023). Only two studies concentrated on the implementation of MRs by pharmacists in primary care (Kwint et al., 2014; Mestres Gonzalvo et al., 2014). In the first study, a research group comprising 49 participants with expertise in MRs sought to pinpoint relevant covariates influencing the quality of MR. These covariates were subsequently rated on a 10-point scale (Mestres Gonzalvo et al., 2014). Our study, in contrast, employed a more comprehensive scoring system to evaluate MR3 reports, providing more detailed results. The second study involved a comparison of the number of DRPs identified in MRs conducted by community pharmacists and expert reviewers (Kwint et al., 2014). A distinctive aspect of our study was the comprehensive analysis, which was slightly broader than the DRPs examined in the previously published study. For instance, we also aimed to take into account elements like the use of medication boxes and the correct application of resources and tools.

The inherent complexity and potential quality issues of interventions like MRs contribute to the lack of clarity in research findings regarding their efficacy and suitability or transferability for various contexts (Alharthi et al., 2022; Nesbit et al., 2022; Robberechts et al., 2024). Therefore, this research may serve as a resource for future quality assessment and control of MR3s facilitating continuous improvement in the quality in addition to effectiveness research of MR3s (Robberechts et al., 2023a). Additionally, the implementation process involves various factors, demanding time, continuous learning, endorsement from patients and healthcare professionals, and fair remuneration (Niquille et al., 2010; Imfeld-Isenegger et al., 2020; Robberechts et al., 2021).

Additionally, it is essential to devise a method to assess the quality of MR3 elements that were not explicitly addressed in the MR3 report, but were nevertheless deemed important by many in our previous research (Robberechts et al., 2023a). It is crucial to differentiate between elements that are often missed, which can be efficiently handled with a checklist or enhanced report templates, and those that are not straightforward to measure of evaluate from the reports. For the latter category incorporating PROMs and PREMs can be used to comprehensively evaluation or MR3 quality (Glenwright et al., 2023). Without a doubt, this would require substantial effort and resources, and it is an area that calls for more detailed scrutiny and dedicated research.

Other open research questions include the impact of the number of completed MR3s on quality, the optimal report template for MR3s, and the additional training needed to improve pharmacists’ MR3 performance.

## 5 Conclusion

This study demonstrates the applicability of quality criteria, established in our previous research, in evaluating the quality of MR3s conducted by community pharmacists. It highlights the crucial role of practical considerations in MR3 implementation, such as a structured report template, phone feedback opportunities, and ongoing pharmacist training. The findings of this study pave the way for internal, peer, and external evaluation of MR3s quality. A comprehensive evaluation of MR3 quality is essential to ensure fidelity in implementation and enable large-scale outcome studies of this valuable pharmaceutical care service.

## Data Availability

The datasets presented in this study can be found in online repositories. The names of the repository/repositories and accession number(s) can be found below: https://doi.org/10.6084/m9.figshare.24884538.v1.
