# Supplementary material for: Use of the BRANT-MERQS scoring table for the quality assessment of type 3 medication review in patients with rheumatoid arthritis and those with type 2 diabetes mellitus
Source: Front Pharmacol. 2024 Aug 16;15:1359568. doi: 10.3389/fphar.2024.1359568 (PMC11362585; doi:10.3389/fphar.2024.1359568)
Supplement: Supplementary file 1 [file DataSheet1.pdf]

Supplementary Material

Use of a scoring table for the quality assessment of type 3 medication review in patients with rheumatoid arthritis or type 2 diabetes mellitus

1 The distribution of the scores

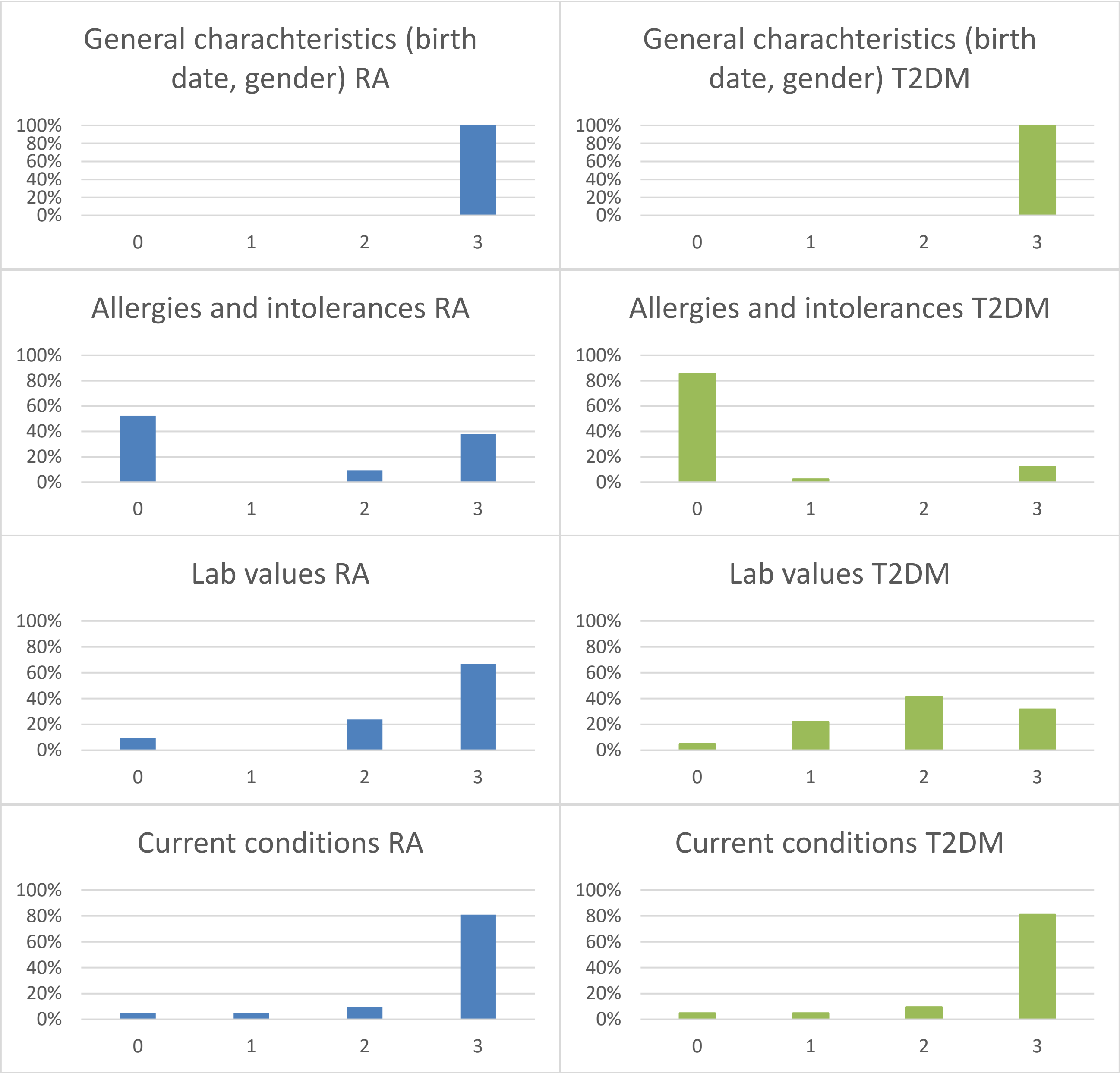

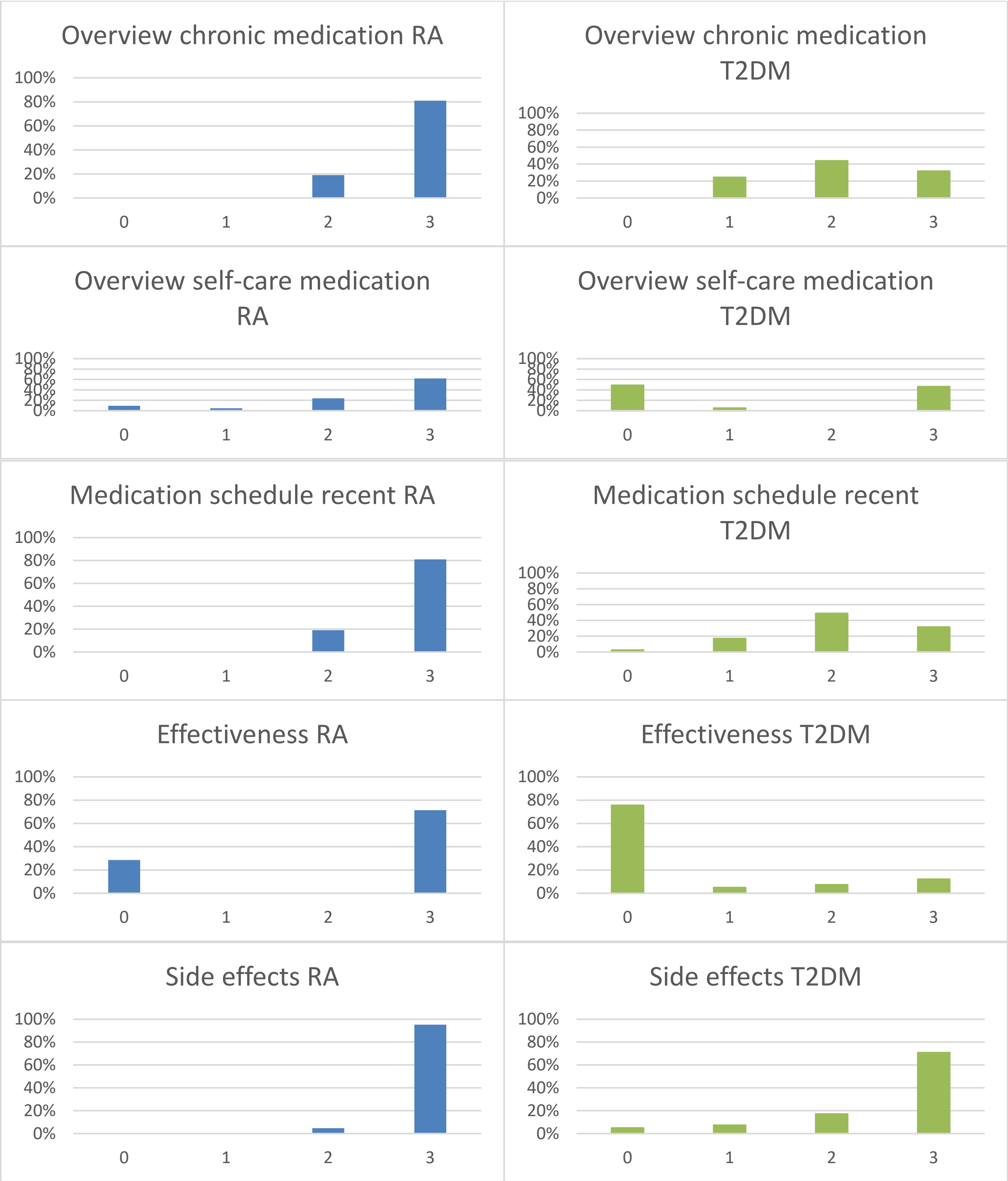
Overview chronic medication RA
100%
80%
60%
40%
20%
0%
0
1
2
3
Overview chronic medication T2DM
100%
80%
60%
40%
20%
0%
0
1
2
3
Overview self-care medication RA
100%
80%
60%
40%
20%
0%
0
1
2
3
Overview self-care medication T2DM
100%
80%
60%
40%
20%
0%
0
1
2
3
Medication schedule recent RA
100%
80%
60%
40%
20%
0%
0
1
2
3
Medication schedule recent T2DM
100%
80%
60%
40%
20%
0%
0
1
2
3
Effectiveness RA
100%
80%
60%
40%
20%
0%
0
1
2
3
Effectiveness T2DM
100%
80%
60%
40%
20%
0%
0
1
2
3
Side effects RA
100%
80%
60%
40%
20%
0%
0
1
2
3
Side effects T2DM
100%
80%
60%
40%
20%
0%
0
1
2
3

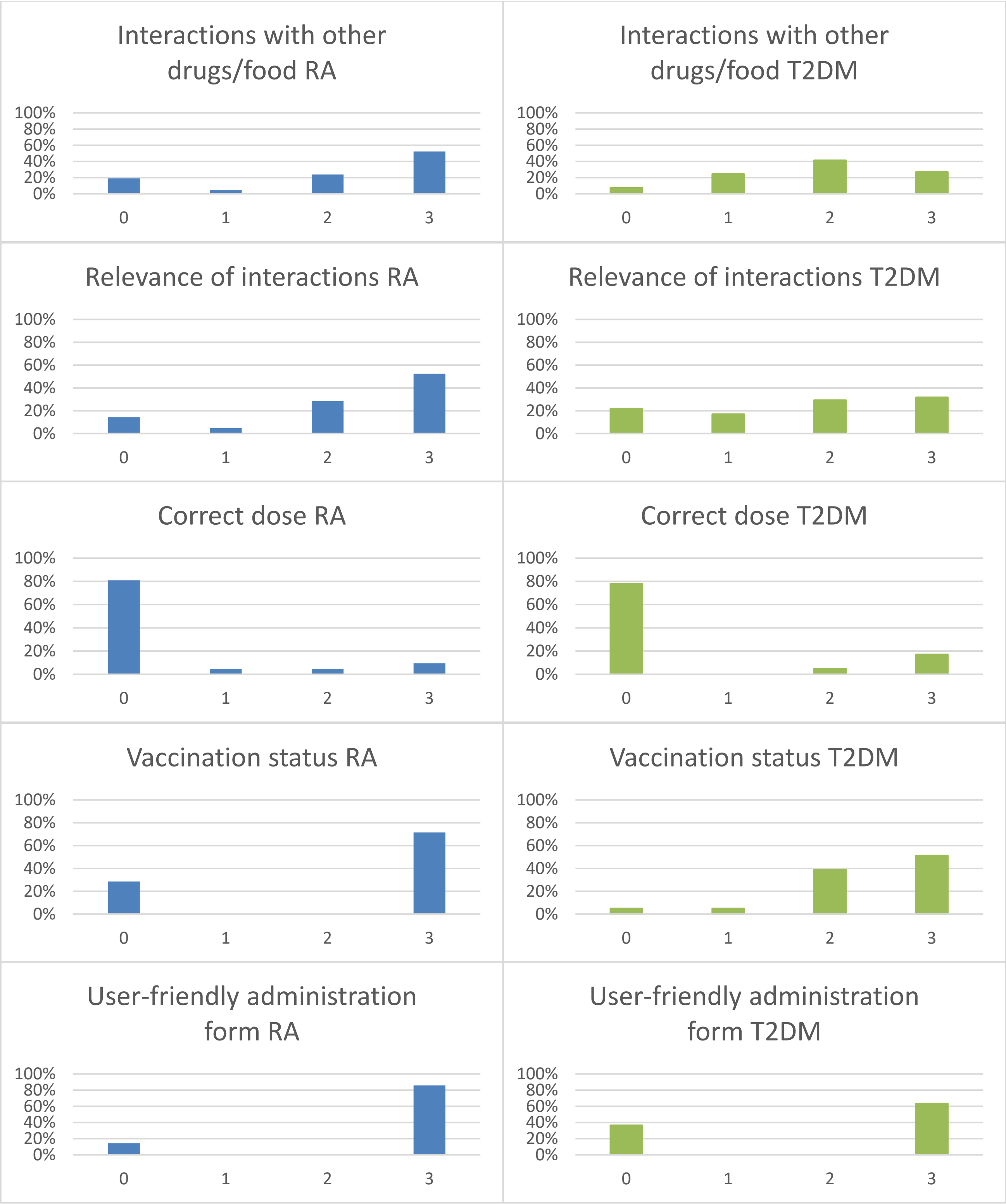
Interactions with other drugs/food RA
Interactions with other drugs/food T2DM
Relevance of interactions RA
Relevance of interactions T2DM
Correct dose RA
Correct dose T2DM
Vaccination status RA
Vaccination status T2DM
User-friendly administration form RA
User-friendly administration form T2DM
100%
80%
60%
40%
20%
0%
0
1
2
3

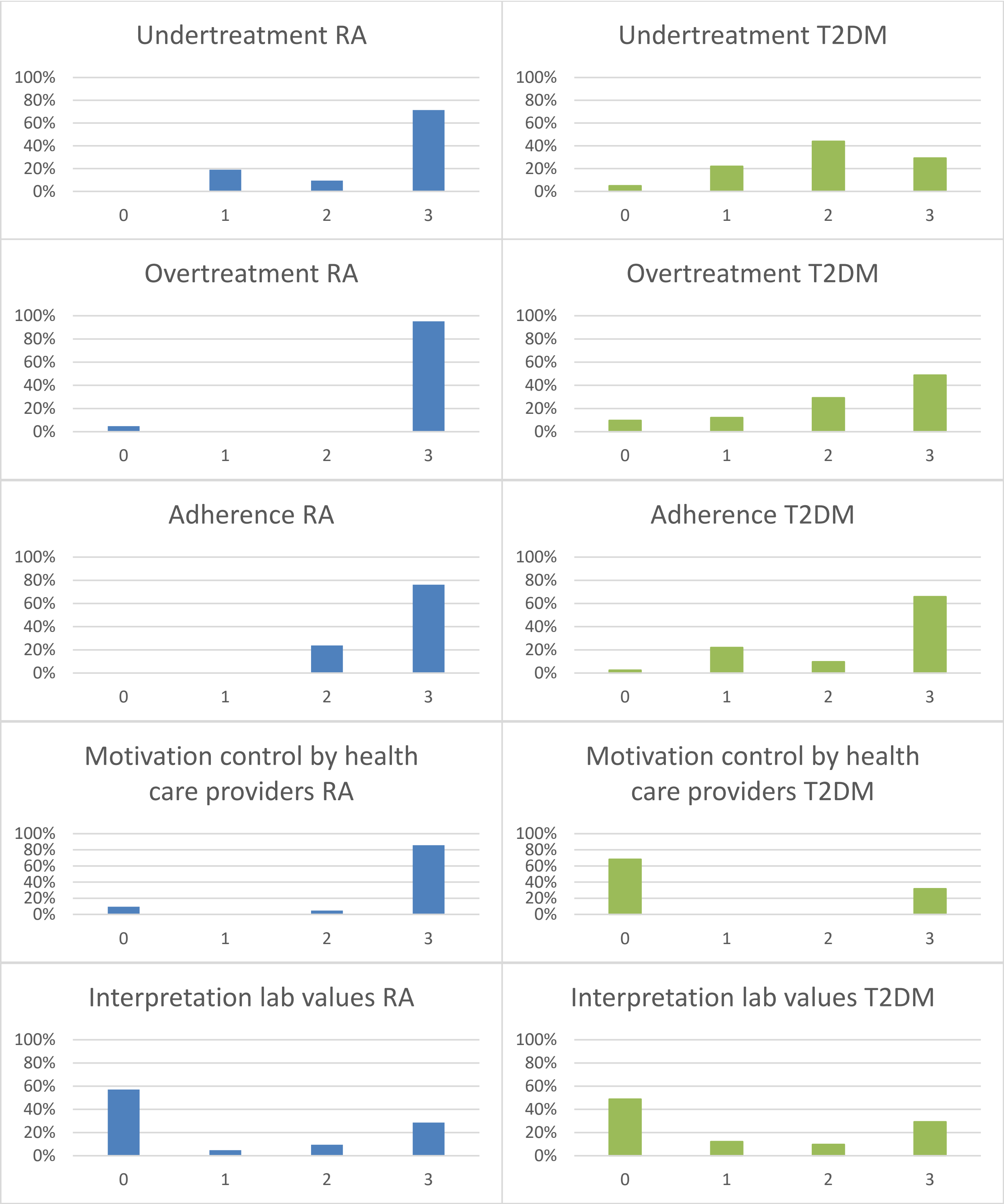
Undertreatment RA
Undertreatment T2DM
Overtreatment RA
Overtreatment T2DM
Adherence RA
Adherence T2DM
Motivation control by health care providers RA
Motivation control by health care providers T2DM
Interpretation lab values RA
Interpretation lab values T2DM
100%
80%
60%
40%
20%
0%
0
1
2
3

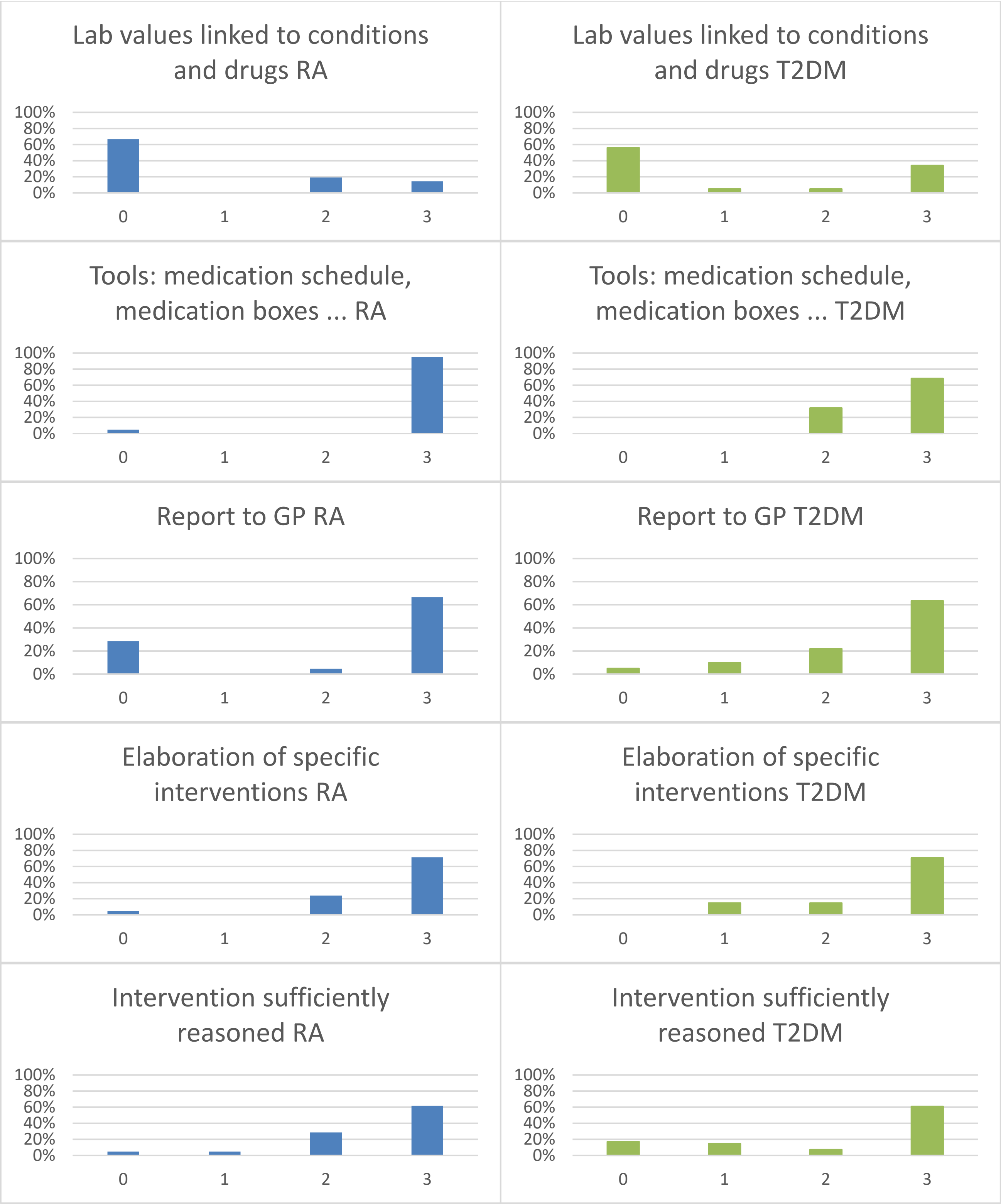
Lab values linked to conditions and drugs RA
Lab values linked to conditions and drugs T2DM
Tools: medication schedule, medication boxes ... RA
Tools: medication schedule, medication boxes ... T2DM
Report to GP RA
Report to GP T2DM
Elaboration of specific interventions RA
Elaboration of specific interventions T2DM
Intervention sufficiently reasoned RA
Intervention sufficiently reasoned T2DM
100%
80%
60%
40%
20%
0%
0
1
2
3

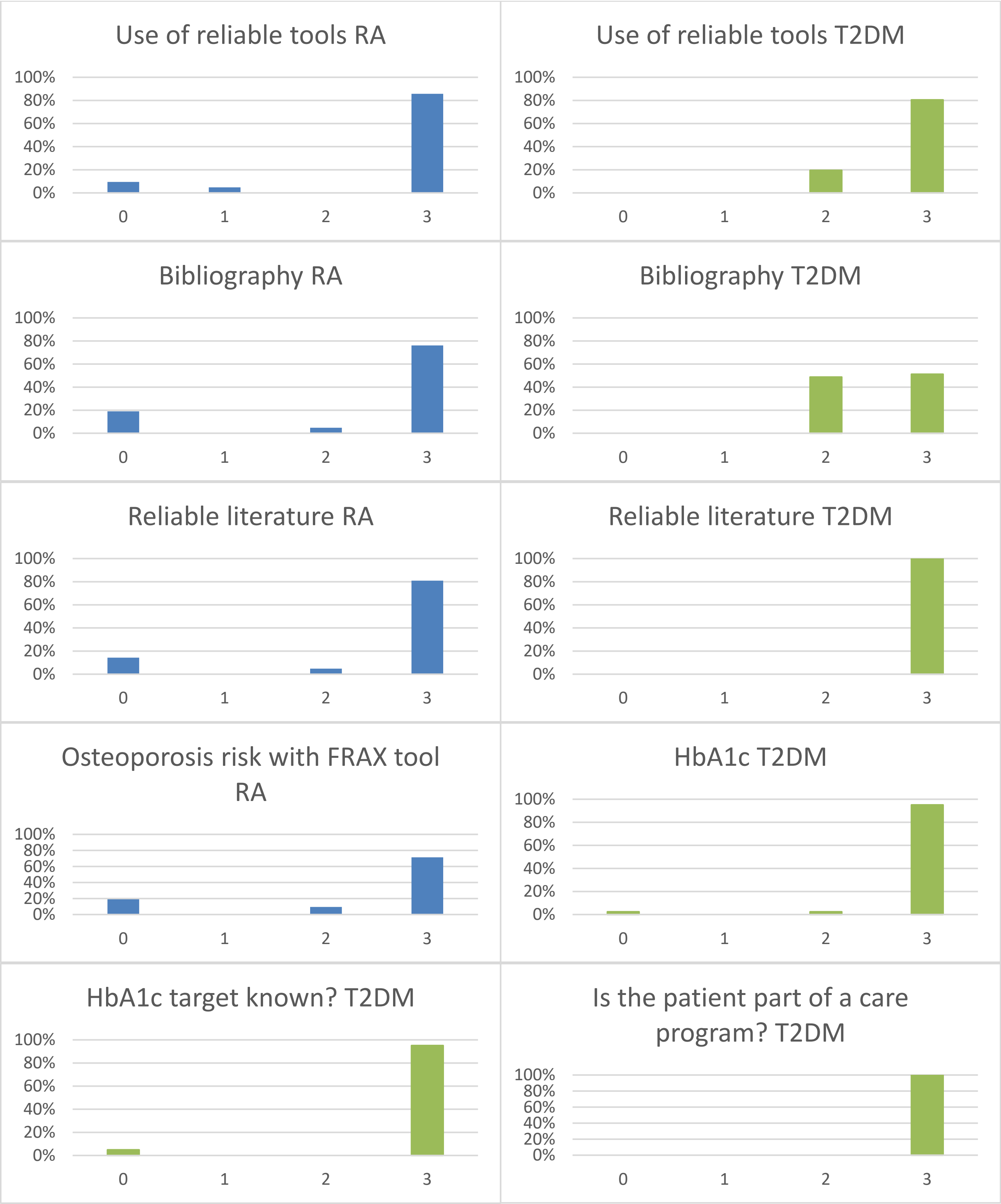

2 Google Form template (translated version)

Questions 16-21 were specifically introduced for the T2DM subproject, while no particular questions were assigned to the RA subproject.

1. Gender of the patient
2. Date of birth of the patient
3. Living conditions of the patient
4. Medical history
5. Medication schedule BEFORE the medication review. Anonymise patient’s and doctor’s data
6. Medication history until 2 years before the medication review. Anonymise patient’s and doctor’s data
7. Clinically relevant data (including date of sampling)
8. Extra general info (vaccination, care programme, …)
9. Patient follow-up: by which healthcare providers is the patient followed up and with what regularity?
10. Why did you suggest a MR tot his patient? What selection criteria were met?
11. What are the patient's concerns that came up during the interview?
12. Are there side effects that the patient suffers from? If yes, which one(s)?
13. Are there relevant interactions? If yes, which one(s)?
14. Are there potential overtreatments? If yes, which one(s)?
15. Are there potential undertreatments? If yes, which one(s)?
16. In your opinion, is the patient eligible for a care programme?
17. If the patient is eligible for one, is he/she actually in that programme?
18. What is the patient's HbA1c value before the medication review? Please add the unit.
19. Does the patient know what his guide values of HbA1c are? If yes, what are they? Please include the unit.
20. Is the patient eligible for a combination preparation so that the complexity of his drugs would be reduced? If yes, what can be replaced by which preparation?
21. How is the patient's self-monitoring? Does he use enough test strips/lancets ...? Explain briefly.
22. Load your excel file of the calculation of the adherence here. Anonymise the patient’s and doctor’s data.
23. For the chronic medications where good adherence is required, adherence is never good – sometimes good – mostly good – always good.
24. For which medications is adherence substandard?
25. The patient has problems taking the medication (think of blister opening, injections, tablet too big, bad taste, anxiety, side effects, does not know indication well enough)
26. The patient uses the following devices for taking his medicines:
    - Medication schedule
    - Individual medication preparation
    - Pill box
    - Other: ………

27. In the pharmacy, we have already had the following conversations with the patient:
    - GGG asthma
    - GGG diabetes
    - Other: ………
    - None of the above

28. What 'problem(s)' will be tackled? Make a priority list of the 'problem(s)' that will be tackled.
29. Also note for each 'problem' how it will be tackled and who will follow it up.
30. What 'problem(s)' will not be addressed until later? Who is responsible for the follow-up?
31. What digital resources did you use?

32. Which textbooks did you use?
33. What screening tools did you use?
34. Load the adapted medication schedule here. Anonymise the patient's and doctor's data.

3 Google Form template (original version)

1. Geslacht patiënt
2. Geboortedatum patiënt
3. Woonsituatie patiënt
4. Medische voorgeschiedenis
5. Medicatieschema VOOR aanvang van de medication review. Anonimiseerde de patiënten- en artsengegevens.
6. Medicatiehistoriek tot 2 jaar VOOR aanvang van het gesprek binnen de medication review. Anonimiseerde de patiënten- en artsengegevens.
7. Klinisch relevante gegevens (incl. datum van de bloedafname), mag evt ook door bestand te uploaden, zie volgende vraag.
8. Extra algemene informatie (bv. vaccins, zorgtrajecten ...)
9. Opvolging patiënt: door welke zorgverleners wordt de patiënt opgevolgd en wat is de regelmaat hiervan.
10. Waarom heb je aan deze patiënt voorgesteld om een MR uit te voeren? Welke selectiecriteria werden er voldaan?
11. Wat zijn de bezorgdheden van de patiënt die tijdens het gesprek naar voor kwamen?
12. Zijn er nevenwerkingen waar de patiënt last van heeft? Indien ja, welke?
13. Zijn er relevante interacties? Indien ja, welke?
14. Zijn er mogelijke overbehandelingen? Indien ja, welke?
15. Zijn er mogelijke onderbehandelingen? Indien ja, welke?
16. Komt de patiënt volgens jou in aanmerking voor "voortraject diabetes"; "zorgtraject diabetes" of "diabetesconventie"?
17. Indien de patiënt in aanmerking komt voor een van de trajecten, zit hij/zij daadwerkelijk in dat traject?
18. Wat is de HbA1c waarde van de patiënt voor de medication review? Geef de eenheid erbij.
19. Weet de patiënt wat zijn richtwaarden van HbA1c zijn? Indien ja, wat zijn ze? Geef de eenheid erbij.
20. Komt de patiënt in aanmerking voor een combinatiepreparaat zodat de complexiteit van zijn geneesmiddelen zou minderen? Indien ja, wat kan vervangen worden door welk preparaat?
21. Hoe is de zelfcontrole van de patiënt? Gebruikt hij voldoende teststrips/lancetten ...? Leg kort toe.
22. Laat hier je het excelbestand 'bereken de therapietrouw' op. Anonimiseerde de patiënten- en artsengegevens.
23. Voor de chronische medicatie waar goede therapietrouw vereist is, is de therapietrouw:
24. Voor welke geneesmiddelen is de therapietrouw ondermaats?
25. De patiënt heeft problemen met de inname van volgende medicatie (denk hierbij aan blister openen, inspuitingen, te grote tablet, slechte smaak, angst, bijwerkingen, kent indicatie niet goed genoeg)
26. De patiënt gebruikt volgende hulpmiddelen voor de inname van zijn geneesmiddelen:
    - Medicatieschema
    - IMV (individuele medicatievoorbereiding)
    - Pillendoos
    - Andere: ………

27. In de apotheek hebben we reeds volgende gesprekken gehad met de patiënt:

- GGG astma
- GGG diabetes
- Andere: ....
- Geen van bovenstaande

28. Welk 'probleem/problemen' zullen aangepakt worden?
29. Noteer hier een prioriteitenlijstje van de aan te pakken 'problemen'? Noteer ook per 'probleem' hoe het aangepakt zal worden en wie het opvolgt.
30. Welk 'probleem/problemen' zullen aangepakt worden?
31. Welke digitale bronnen heb je gebruikt?
32. Welke handboeken heb je gebruikt?
33. Welke screeningstools heb je gebruikt?
34. Laat hier je het aangepast medicatieschema op. Anonimiseerde de patiënten- en artsengegevens.

4 Word template (translated version)

Report medication review (initials + date)

Patient data: (unique number)	Male/female:
Living conditions:	Date of birth:
Confidential counselor:	

**Medication**	**Indication**	**Relevant clinical data (+ date of values)**

The precise medication schedule is enclosed.

Allergies:

Care programmes:

Initiation of conversation

Concerns of the patient

Adherence to therapy (excel file attached)

1. Patient adherence, based on the medication delivered is generally:

   Never good - sometimes good - usually good - always good

   Comments:

2. The patient has problems taking the following medication
   - Drug a
   - Drug b
   - Reason: blister opening, injections, tablet too big, bad taste, anxiety, side effects, does not know indication well enough

3. In the pharmacy, we have already had the following conversations with the patient
   - ☐ GGG asthma
   - ☐ GGG diabetes
   - ☐ Other, namely:
   - ☐ None of the above

4. The patient uses the following tools from the pharmacy:
   - ☐ Medication schedule
   - ☐ Individual drug preparation
   - ☐ Pill box
   - ☐ Other, namely:

Relevant drug interactions

Drug interaction 1

- Drug:
  Explanation:

Drug interaction 2

- Drug:
  Explanation:

Adverse effects suffered by the patient

Possible overtreatment

Possible undertreatment

Further appointments with treating doctor(s)

Priority	Action point	Who does the follow-up?	By when?

5 Word template (original version)

Verslag medicatiebeoordeling (initialen + datum)

Patiëntgegevens: (uniek nr) Man/vrouw:

Woonsituatie: Geboortedatum:

Vertrouwenspersoon:

**Medicatie**	**Indicatie**	**Relevante klinische gegevens (datum van de waarden)**

In bijlage kan u het accurate medicatieschema vinden.

Allergieën:

Zorgtrajecten:

Aanleiding gesprek

Bezorgdheden van de patiënt

Therapietrouw (excelbestand in bijlage)

5. De therapietrouw van de patiënt, op basis van de afgeleverde medicatie is in het algemeen:

   Nooit goed – soms goed – meestal goed – altijd goed

   Opmerkingen:

6. De patiënt heeft problemen met de inname van volgende medicatie
   - Geneesmiddel a
   - Geneesmiddel b
   - *Reden: blister openen, inspuitingen, te grote tablet, slechte smaak, angst, bijwerkingen, kent indicatie niet goed genoeg*

7. In de apotheek hebben we reeds volgende gesprekken met de patiënt gehad
   - ☐ GGG astma
   - ☐ GGG diabetes
   - ☐ Andere, namelijk:
   - ☐ Geen van bovenstaande
   -
8. De patiënt gebruikt volgende hulpmiddelen uit de apotheek:
   - ☐ Medicatieschema
   - ☐ IMV (Individuele medicatievoorbereiding)
   - ☐ Pillendoos
   - ☐ Andere, namelijk:
   -

Relevante interactie

Interactie 1

- Geneesmiddelen:

  Uitleg:

Interactie 2

- Geneesmiddelen:

  Uitleg:

Ongewenste effecten waar de patiënt last van heeft

Mogelijke overbehandeling

Mogelijke onderbehandeling

Verdere afspraken met behandelende arts(en)

Prioriteit	Actiepunt	Wie volgt het op?	Tegen wanneer?
